# Development and Evaluation of the Personal Patient Profile–Bladder Cancer (P3-BC): A Web-Based Decision Support System for Patients Considering Cystectomy and Urinary Diversion

**DOI:** 10.3390/cancers18101501

**Published:** 2026-05-07

**Authors:** Nihal E. Mohamed, Donna L. Berry, Justin McReynolds, Talia Korn, Holden Kata, Emma Benn, Danielle Scharp, John Sfakianos, Reza Mehrazin, Peter Wiklund, William B. Lober

**Affiliations:** 1 Department of Urology, Icahn School of Medicine at Mount Sinai, New York, NY 10029, USA; holden.kata@mountsinai.org (H.K.); john.sfakianos@mountsinai.org (J.S.); reza.mehrazin@mountsinai.org (R.M.); peter.wiklund@mountsinai.org (P.W.); 2 School of Nursing, University of Washington, Seattle, WA 98195, USA; donnalb@uw.edu (D.L.B.); mcjustin@uw.edu (J.M.); lober@uw.edu (W.B.L.); 3 Department of Population Health Science and Policy, Icahn School of Medicine at Mount Sinai, New York, NY 10029, USA; talia.korn@mountsinai.org (T.K.); emma.benn@mountsinai.org (E.B.); 4 Division of General Internal Medicine, Icahn School of Medicine at Mount Sinai, New York, NY 10029, USA; danielle.scharp@mountsinai.org

**Keywords:** bladder cancer, cystectomy, urinary diversion, shared decision-making, decision support, patient education

## Abstract

**Background/Objectives:** Radical cystectomy with urinary diversion is the standard treatment for patients with muscle-invasive bladder cancer, which represents a complex, preference-sensitive decision about the diversion options. To facilitate patient-clinician shared decision-making, we developed and evaluated the feasibility and acceptability of the Personal Patient Profile–Bladder Cancer (P3-BC), a web-based decision support system for patients considering urinary diversion options. **Materials and Methods:** We employed an iterative development approach following an established framework, including qualitative assessments of radical cystectomy patients’ (n = 30) needs to inform content development, followed by usability testing with 10 key stakeholders. We then conducted a feasibility study with patients newly diagnosed with bladder cancer undergoing cystectomy. Feasibility was assessed through P3-BC completion rates among patients randomized to the intervention and study retention rates. Acceptability was measured using a program-based 12-item questionnaire integrating the six-item Acceptability E-Scale. Secondary outcomes included decisional conflict, shared decision-making, psychological distress, and decisional regret. **Results:** Of 114 eligible patients, 24 provided consent (21% consent rate), and 15 were randomized to receive the P3-BC intervention (n = 10) or usual care (n = 5). Retention was 93% at 1 month and 73% at 3 months. Among intervention participants, P3-BC achieved high feasibility (100% accessed the program) and acceptability, with 86% reporting overall satisfaction and 86% reporting ease of use. No statistically significant between-group differences were observed in secondary outcomes. Although the study was not powered to examine group differences, numerical trends suggest improvements in decisional conflict and symptom burden over time, particularly in the intervention group. **Conclusions:** P3-BC demonstrated feasibility and acceptability as a web-based decision support intervention for patients with bladder cancer considering cystectomy and urinary diversion. Primary barriers to enrollment included treatment burden, limited diagnosis-treatment time, and technology concerns. Findings support progression to an adequately powered randomized controlled trial to evaluate clinical effectiveness.

## Simple Summary

Cystectomy (surgery to remove the bladder) often requires patients to choose between different methods of urinary reconstruction; a life-changing decision that affects daily function, body image, and quality of life. Yet, many patients receive limited support while making this choice. To address this gap, the research team developed a web-based tool called the Personal Patient Profile–Bladder Cancer (P3-BC) to help patients understand their options, clarify their personal values, and make more informed decisions. The tool was first built using feedback from 30 patients and 10 healthcare stakeholders, then tested in a small pilot study with 15 patients. All patients who used it completed the program, and most found it easy to use and helpful. While the study was small, early results are promising and support testing the tool in a larger study. These findings lay the groundwork for a larger trial examining the effect of P3-BC on treatment decisions.

## 1. Introduction

Bladder cancer represents a significant public health burden, with an estimated 82,290 new cases diagnosed annually in the United States [1]. About 25–30% of patients present with muscle-invasive bladder cancer (MIBC; T2-T4a), for which radical cystectomy with urinary diversion is the standard treatment [2]. Radical cystectomy is also recommended for patients with high-risk, non-MIBC who are unresponsive to treatment [3].

Radical cystectomy involves complex decisions regarding the selection of the urinary diversion method. Urinary diversion methods include: (1) ileal conduit, (2) continent cutaneous reservoir, and (3) orthotopic neobladder. Each method involves distinct surgical complexity and lifestyle implications [4,5,6,7]. Ileal conduits require a stoma with continuous external drainage; continent reservoirs allow intermittent catheterization; and neobladders enable volitional voiding [4,5,6,7]. The decisions regarding the diversion options depend on cancer stage, comorbidities, functional capacity, lifestyle, body image, and patient values regarding urinary control and sexual function [8,9,10,11,12,13]. Complications vary considerably across diversion types, with stoma-related issues affecting up to 31% of ileal conduit patients (e.g., parastomal dermatitis, parastomal hernia, and stomal stenosis) [14], and neobladders and continent reservoirs having complication rates of 3–7% and 13–30%, respectively (e.g., urinary tract infections, ureteric strictures, and metabolic acidosis) [15,16].

Despite guideline recommendations from the American Urological Association (AUA), European Association of Urology (EAU), and American Society of Clinical Oncology (ASCO) advocating for shared decision making (SDM) and multidisciplinary discussions, implementation gaps persist. Although more than 70% of clinicians prefer SDM, actual use may occur in only 10% of encounters [17,18,19]. Consequently, patients frequently report unmet informational and decisional needs regarding urinary diversion–related quality of life [20,21]. Factors beyond survival, such as sexual function, independence, and avoidance of ostomy appliances, strongly influence clinical decisions [22,23,24]. Studies suggest variation in SDM, with patients undergoing cystectomy alone reporting less SDM than those receiving trimodal therapy [25]. Further, patients frequently report dissatisfaction with the quantity and quality of information provided [26,27].

Decision support tools can facilitate SDM by providing comprehensive information, clarifying values, and supporting communication [28,29]. While decision support tools exist in the context of prostate and renal cancer, such tools are lacking in the setting of bladder cancer [30]. One prior study evaluated a bladder cancer decision support tool, finding it acceptable but needing further validation [31]. Web-based decision support tools are advantageous for tailoring content, presenting probabilistic information, incorporating multimedia testimonials, and offering values clarification exercises [32,33]. Older adults, who comprise most bladder cancer patients, increasingly use the internet, suggesting that web-based interventions may be feasible for this population [34].

This study was guided by O’Connor’s Decision Support Framework, which outlines key decisional determinants, informs tailored interventions, and provides a structure for evaluating decision support outcomes [35]; and the Assessment, Decision, Adaptation, Production, Topical Experts, Integration, Training, Testing (ADAPT-ITT) framework, which supports systematic adaptation of evidence-based interventions [36]. These frameworks informed our adaptation of an existing decision aid we developed for early-stage prostate cancer patients (Personal Patient Profile–Prostate Cancer; P3-P) for patients with bladder cancer considering radical cystectomy (P3-BC), maintaining fidelity while tailoring content to the clinical and psychosocial context [37,38].

The primary objective of this study was to adapt and test P3-BC, a tailored web-based decision support system designed to facilitate SDM about urinary diversion options among bladder cancer patients undergoing radical cystectomy. Thus, we aimed to: (1) develop system specifications and use cases, (2) design application architecture and content, (3) assess P3-BC usability, and (4) evaluate feasibility and acceptability in a clinical pilot study.

## 2. Materials and Methods

### 2.1. Study Design

This study was conducted at a single academic medical center using a sequential, iterative development and evaluation design guided by O’Connor’s Decision Support Framework and the ADAPT-ITT framework [36]. The study received Institutional Review Board approval and followed established guidance for developing and evaluating decision support interventions, including the International Patient Decision Aid Standards (IPDAS) [39]. The ADAPT-ITT framework consists of eight phases (i.e., Assessment, Decision, Adaptation, Production, Topical experts, Integration, Training, and Testing) providing a structured approach to adapt existing interventions to new clinical contexts while preserving core theoretical components [36].

P3-BC was adapted from the P3-P decisional support tool in four overarching stages encompassing the eight phases of the ADAPT-ITT framework (Supplementary Figure S1): (1) formative research and content development (Assessment, Decision), (2) system architecture and design (Adaptation, Production, Topical experts), (3) pre-pilot usability testing (Integration, Training), and (4) clinical pilot testing (Testing).

### 2.2. Stage 1: Formative Research and Content Development

In the Assessment phase, we conducted formative research to characterize decision-making needs among patients undergoing radical cystectomy [19,21]. Adults aged ≥18 years with MIBC or treatment-unresponsive non-MIBC for whom cystectomy was recommended were recruited from the urology clinic. Eligible participants were those who could provide informed consent and participate in interviews. A total of 30 patients who had undergone radical cystectomy participated in this phase. Discussions explored informational needs, decisional priorities, concerns about diversion options, and preferences for decision support delivery. Interviews were audio-recorded, transcribed verbatim, and analyzed using iterative content analysis to identify key informational gaps, decision processes, and support needs.

In the Decision phase, the legacy P3-P program, developed by our team and found to reduce decisional conflict among men with localized prostate cancer [37,38], was reviewed in detail by the multidisciplinary team. The team systematically compared prostate and bladder cancer decisional contexts. Similarities supporting adaptation included: multiple treatment options, preference-sensitive trade-offs, need for explicit values clarification, and an older adult target population weighing survival, function, and lifestyle. The team concluded that adapting P3-P would be more efficient and clinically relevant than de novo development while allowing for substantial content modification to address the unique bladder cancer context.

### 2.3. System Architecture and Design

In the Adaptation phase (Supplementary Tables S1 and S2), a structured adaptation matrix specified which P3-P components were retained, modified, or newly developed for P3-BC. During the Production phase, new and revised materials were developed based on formative findings. P3-BC content included: (1) detailed descriptions and illustrations of ileal conduit, continent cutaneous reservoir, and orthotopic neobladder, (2) probabilistic information on perioperative outcomes, complications, and quality-of-life domains presented via interactive graphics, (3) video testimonials depicting daily self-care and lifestyle with each diversion, (4) values clarification exercises addressing urinary control method, body image, physical activities, sexual function, independence, and lifestyle, and (5) communication coaching to prepare patients for consultations, and (6) curated links to high-quality external resources.

In the Topical Experts phase, content underwent review by a multidisciplinary team that included urologic oncologists, ostomy and wound care nurses, sexual health counselors, health informaticists, web designers, health literacy specialists, instructional designers, and patient advisors. Medical content was validated against contemporary guidelines from the American Urological Association, European Association of Urology, and American Society of Clinical Oncology [2,17,18]. Patient advisors who had undergone cystectomy with different diversions reviewed materials for clarity, relevance, and emotional tone.

In the production phase, P3-BC was structured as six sequential modules driven by a tailoring algorithm. Module 1 (Structured Baseline Survey) captured demographics, clinical history, psychosocial factors, values and priorities, preferred decision-making role, and information preferences. Module 2 (Tailored Educational Content) provided individualized pathways through urinary diversion-specific information, emphasizing content areas aligned with each patient’s reported concerns. Module 3 (Video Testimonials) featured short first-person narratives from three patients representing each diversion type, depicting practical lifestyle considerations to support realistic outcome expectations. Module 4 (Values Clarification Exercises) included structured, rating-scale exercises to help patients weigh trade-offs across the diversion options. Module 5 (Communication Coaching) provided example questions and preference statements patients could use during surgical consultations, with language tailored to each patient’s prioritized values. Module 6 (Curated Resources) provided links to vetted external resources from the American Urological Association, the Bladder Cancer Advocacy Network (BCAN), and the United Ostomy Association of America. Following program completion, a summary of the patient’s recorded preferences and values was shared by research staff with the treating physician prior to the treatment consultation, to facilitate individualized SDM discussions. Representative screenshots of P3-BC are provided in Supplementary Figures S2 and S3.

P3-BC was implemented as a stand-alone, web-based application accessible via desktop computer and smartphone. The system used an assessment-driven tailoring algorithm. Following consent, all study participants first completed a P3-BC Web-based, structured baseline survey capturing demographic characteristics, clinical factors, psychosocial factors, values and concerns, preferred decision-making role, and information preferences. Patients in the control group exit the program after completing the baseline survey. Tailored pathways emphasized content aligned with individual concerns and priorities. P3-BC recorded preferences and values of the intervention group were shared with the physicians before treatment consultation by the research staff to facilitate treatment discussions and SDM.

### 2.4. Pre-Pilot Usability Testing

In the Integration phase, formative research findings, expert recommendations, patient advisor feedback, technical input, health literacy guidance, and guideline-based evidence were synthesized into a cohesive intervention. The Training phase combined staff preparation with pre-pilot usability testing. Ten stakeholders (including 4 patients who had completed bladder cancer treatment ≥ 6 months earlier, 4 patient advocates, a social worker, and an oncology nurse) participated in usability testing. Using a think-aloud protocol [40], participants completed P3-BC while verbalizing their thoughts and difficulties. Research staff documented navigation problems, unclear content, technical issues, helpful features, suggestions, and overall impressions.

Pre-pilot testing identified content, navigation, technical, and presentation issues. Revisions included simplifying medical terminology, revising ambiguous value questions, enhancing explanations of surgical procedures, improving progress indicators and navigation functionality, addressing technical issues, and improving visual design elements.

### 2.5. Clinical Pilot Feasibility Testing

Following pre-pilot revisions, a clinical pilot was conducted in the urology clinic. Patients with a recent MIBC diagnosis or high-risk non-MIBC unresponsive to treatment scheduled for consultation regarding cystectomy and urinary diversion were eligible. Exclusion criteria were inability to understand English, severe cognitive impairment precluding informed consent or program use, and insufficient time before surgery to complete the intervention. Potential participants were identified via clinic schedules and medical record review, approached by research staff, and provided written informed consent.

Participants were randomized to receive the intervention (P3-BC) or usual care after completing the program’s Web-based baseline survey. Intervention participants completed P3-BC in a private clinic room before meeting the urologist or at home via website access. A research assistant was available for technical support, but did not influence interaction with the intervention. The system automatically recorded completion time, navigation patterns, and patient preferences and values.

### 2.6. Primary Outcome Measures

Feasibility was assessed through program completion rate and study retention rates. Acceptability was assessed in two ways. First, participants completed the six-item Acceptability E-Scale validated by Tariman et al. (2011) for web-based patient-reported outcomes in cancer care [41] along with six additional study-specific items (12 items total) at the end of program navigation. These items were embedded within the P3-BC website and assessed ease of use, understandability, helpfulness, satisfaction, and perceived value of specific program components using a 5-point Likert scale (1 = completely disagree/not acceptable, to 5 = completely agree/acceptable), with higher scores indicating greater acceptability. Feasibility was evaluated based on recruitment (consent rate), retention (follow-up completion rates), and program completion rates. Second, a 1-month follow-up questionnaire included 11 additional items using a 6-point Likert scale to assess participants’ perceptions of study procedures, acceptance of the intervention, and satisfaction with the decision aid.

### 2.7. Secondary Outcome Measures

All secondary outcomes are measured by validated instruments, including the decisional conflict, decisional regret, psychological distress, and SDM validated scales with established psychometric properties in different oncology populations. The Decisional Conflict Scale (DCS; 16 items, α = 0.91) was validated by O’Connor (1995) [42] and is widely used across cancer decision aid trials. The Decision Regret Scale (DRS; 5 items, α = 0.55) was validated by Brehaut et al. (2003) [43] and has been applied extensively in surgical oncology decision-making research. The Brief Symptom Inventory-18 (18 items, α = 0.95) has undergone rigorous validation for psychological distress in cancer settings. The Shared Decision-Making Questionnaire (SDM-Q-9; 9 items, α = 0.90) has been validated in multiple oncology contexts. The outcome measures were assessed at 1 month and 3 months [42,43,44].

### 2.8. Statistical Analysis

Participant characteristics and outcome measures were summarized using descriptive statistics (means, standard deviations, frequencies, and percentages). Between-group comparisons for descriptive statistics at baseline were conducted by Welch’s independent samples t-test for continuous variables and two-sided Fisher’s exact tests for categorical variables. Between-group comparisons for the secondary outcomes were conducted using independent-sample *t*-tests, with Welch’s correction applied, when appropriate, due to unequal sample sizes and variances. All analyses were performed using IBM SPSS statistical software (Version 25.0, Armonk, NY, USA), with *p* < 0.05 considered statistically significant.

## 3. Results

### 3.1. Recruitment and Retention

Of 114 eligible participants, 24 (21%) provided consent. Common reasons for declining participation included disease and treatment burden, limited time, and lack of confidence in using technology independently. Among those who consented, 4 participants were lost to follow-up prior to participation, leaving 20 participants (83%) who initiated the P3-BC program-based baseline survey. Among these participants, 5 did not complete a sufficient proportion of the baseline survey required for randomization and were therefore not randomized.

In total, 15 participants were randomized, with 10 assigned to the P3-BC intervention group and 5 to the usual care control group. Participants were randomized in a 2:1 ratio (intervention:control) to maximize data yield from the intervention arm. Randomization occurred after completion of baseline assessments. Given the small pilot sample, randomization was intended to support preliminary feasibility evaluation rather than to ensure balance across baseline characteristics. Retention was high, with 14 participants completing the 1-month follow-up survey (93%; 10 intervention, 4 control) and 11 participants completing the 3-month follow-up survey (73%; 8 intervention, 3 control).

### 3.2. Participant Characteristics

Among the total study sample (n = 15), the mean age was 70 years (SD = 7.1; range 64–84). The intervention group was significantly younger than the control group (67.9 ± 3.1 versus 79.8 ± 4.1 years; t(6.38) = −5.72, *p* = 0.001). The total sample was predominantly White (86%), non-Hispanic (100%), male (73%), and married (93%). Common comorbidities included diabetes (40%), heart disease (13%), chronic kidney disease (7%), and peripheral neuropathy (7%). The intervention group included a higher proportion of men (90%) compared with the control group (40%); however, this difference was not significant. Additional sample characteristics are provided in Table 1.

### 3.3. Feasibility Outcomes

Among intervention participants who completed the program, missing data were minimal (0.5% of items). The 21% consent rate reflected recruitment challenges in this population, given the short time period between diagnosis and cystectomy. High P3-BC access rates (100%) and retention among study-enrolled participants (93% at 1 month, 73% at 3 months) demonstrated the feasibility of the P3-BC and the follow-up procedures.

### 3.4. Acceptability Outcomes

The highest-rated aspects of P3-BC were clarity and usability, with 100% of participants rating program navigation and tailoring questions as understandable, and 86% reporting the program was easy to use and expressing overall satisfaction. Informational content was rated highly, particularly on statistics (86% high ratings). Lower ratings were observed for communication coaching (43% high ratings), suggesting areas for refinement (Table 2).

Participants most strongly endorsed P3-BC’s role in decision support (71% agreement), knowledge acquisition, values clarification, technical self-care skills, and anxiety reduction (63% agreement each). Lower endorsement was observed for preparation for surgical side effects (38%) and support for physician communication (50%).

Among respondents with available data (n = 8), 75% indicated they would recommend the program. Program use during consultations was variable, with 38% reporting use of program information during physician consultations. Engagement with other information sources was high, with 88% using and finding other bladder cancer information sources helpful (Table 3).

### 3.5. Secondary Outcomes

No statistically significant differences were observed between intervention and control groups for any secondary outcome at the one or three-month assessments. At one month, SDM scores were similar between groups (5.43 ± 0.54 vs. 5.47 ± 0.42; *p* = 0.889), while Brief Symptom Inventory scores were numerically higher in the intervention group (0.51 ± 0.73 vs. 0.15 ± 0.21; *p* = 0.238).

At three months, decisional conflict scores were slightly lower in the intervention group (4.28 ± 0.39) compared with the control group (4.56 ± 0.39; *p* = 0.354). Psychological distress scores remained numerically higher among intervention participants (0.26 ± 0.32 vs. 0.07 ± 0.08; *p* = 0.160), while decisional regret scores were slightly lower in the intervention group (4.38 ± 0.59 vs. 4.67 ± 0.42; *p* = 0.399).

Within-group trends suggested numerical improvements in decisional conflict and symptom burden from one to three months in both groups. Decisional conflict total mean score decreased from 5.43 to 4.28 in the intervention group and from 5.47 to 4.56 in the control group. Brief Symptom Inventory scores declined from 0.51 to 0.26 in the intervention group and from 0.15 to 0.07 in the control group (Table 4 and Table 5).

## 4. Discussion

This study engaged key stakeholders to adapt a web-based decision support system originally developed for prostate cancer to address critical decisional and informational needs among patients with bladder cancer undergoing radical cystectomy. Findings provide preliminary evidence supporting the feasibility and acceptability of P3-BC and underscore its potential to improve patient shared decision-making.

Study results showed a 21% consent rate, reflecting known recruitment challenges observed in other cancer studies [45]. Low consent rate in our study may also reflect recruitment barriers common in the bladder cancer patient population, including disease burden, limited time between diagnosis and surgery, and technology concerns. These factors may introduce sampling bias and limit the generalizability of supportive care interventions, including P3-BC.

Our results, however, showed that engagement with P3-BC and retention among enrolled participants in this study were excellent. Completion of p3-BC was 100%, and retention rates were 93% at one month and 73% at three months, supporting the feasibility of integrating P3-BC into pre-treatment clinical workflows. High acceptability rates further demonstrate the appropriateness of the P3-BC for an older bladder cancer population. The median age at bladder cancer diagnosis is 74 years, making technology concerns particularly relevant [46]. Older adults with cancer may face barriers to digital health adoption, including low digital literacy, lack of knowledge and skills, concerns about technology access, and uncertainty about digital communication quality [47]. However, studies show that when appropriately designed, older adults can successfully engage with digital interventions [48,49]. Iterative refinements during the pre-pilot phase, such as improvements to navigation and terminology identified through think-aloud testing [40], likely contributed to the favorable outcomes observed in the feasibility pilot study.

The ADAPT-ITT framework provided systematic guidance throughout P3-BC development consistent with its successful application across diverse health interventions. However, application of ADAPT-ITT to cancer patient decision aids remains limited, with most cancer decision aid development relying on literature reviews, focus groups, and pre-existing tools without systematic adaptation frameworks [50,51,52]. The Assessment phase’s extensive formative research (30 patients) identified patient information and decisional needs that shaped content priorities. The Decision phase’s rigorous prostate-bladder cancer comparison justified P3-P adaptation rather than de novo development, while identifying necessary modifications for urinary diversion complexity. The Topical Experts phase elicited multidisciplinary perspectives to ensure clinical relevance and technological robustness, consistent with multi-stakeholder engagement approaches documented in other ADAPT-ITT applications [51,52]. Integration and Training phases’ usability testing revealed issues undetectable by expert review alone, while the Testing phase’s comprehensive outcomes evaluation supports advancement to effectiveness trials. This structured approach preserved P3 architecture fidelity while achieving P3-BC-related bladder cancer context fit.

High acceptability ratings support P3-BC’s patient-centered design. Overall, 100% of intervention group participants found the program navigation questions understandable, 86% found P3-BC easy to use, and 86% expressed overall satisfaction. These ratings likely reflect the careful, pre-deployment attention to content development and are consistent with prior studies showing that ease of use and comprehension are key determinants of patient engagement [50].

Communication coaching received mixed ratings (43% high ratings), suggesting the need for refinement through video demonstration of communication examples. This finding aligns with the broader literature showing variable effectiveness of communication coaching components in decision aids [53]. Seventy-one percent agreed that the program helped them make treatment decisions, with 63% reporting increased knowledge, values clarification, and reduced anxiety, validating the intervention’s core objectives. However, lower ratings for preparation for surgical side effects (38%) and physician communication support (50%) confirm areas for program future enhancement.

Despite guideline recommendations for SDM in urinary diversion selection, no validated tools exist [21,26]. Systematic reviews of other cancer populations with similar decisional needs confirm the impact of decision aids on improving knowledge, increasing values clarity, and patient engagement across conditions [28,29]. P3-BC extends this evidence to bladder cancer’s complex, preference-sensitive decisions involving ileal conduit, continent reservoir, or neobladder reconstruction. P3-BC provided decisional support, and feasibility outcomes supported process success, positioning the P3-BC for an effectiveness evaluation.

The absence of statistically significant between-group differences in secondary outcomes reflects the study’s small sample size and limited statistical power, as expected in this feasibility study. The observed numerical trends suggesting improvements in decisional conflict and symptom burden over time in both groups may reflect natural adaptation processes following diagnosis or beneficial elements of study participation itself. The statistically significant younger age of intervention participants (12-year mean difference) represents an important limitation requiring consideration in future studies by including larger samples, stratified randomization, or covariate adjustment.

### 4.1. Strengths and Limitations

Systematic ADAPT-ITT application [36] ensured methodological rigor, documented transparently for replication. Iterative development with 30 patients, multidisciplinary review, think-aloud testing [40], and clinical piloting followed complex intervention best practices. Dual theoretical grounding in established frameworks enhanced the study design and evaluation rigor. The use of validated measures for outcomes enables cross-study comparisons. Multidisciplinary team involvement and focus on understudied urinary diversion decisions address key gaps.

This study was designed as a feasibility pilot and was not powered to evaluate clinical effectiveness. The small sample size and 21% consent rate should be interpreted in the context of a highly time-sensitive clinical setting, in which patients were asked to consider a complex surgical decision soon after diagnosis. The group difference in age may have influenced intervention engagement and usability outcomes. Future studies should consider stratified randomization to reduce potential baseline imbalance. The small pilot feasibility sample (n = 15) precluded subgroup analyses and limited the generalizability of secondary outcomes. P3-BC remains to be validated for clinical effectiveness; thus, the current results should be interpreted within that context. The single-center design further constrains applicability to community practice settings. The sample was predominantly White, male, and non-Hispanic, which limits the generalizability of the study findings to more diverse populations. There is a need for broader recruitment in future studies to ensure the intervention is evaluated across a wider range of patient experiences and demographic backgrounds [54]. Larger randomized trials are also needed to evaluate the effectiveness of P3-BC in improving treatment outcomes and to assess clinician perspectives. 

### 4.2. Future Directions

Multisite randomized controlled trials should evaluate and formally validate P3-BC for clinical effectiveness, with pre-specified primary outcomes of decisional conflict and values-concordance, before P3-BC effectiveness on knowledge, decisional conflict, values-concordance, and SDM quality, with adequate power for subgroup analyses across demographics and stratified randomization to prevent baseline imbalances. Implementation research should optimize delivery timing/setting, electronic health record integration, and physician acceptability. Intervention adaptation addressing bladder preservation versus cystectomy tradeoffs warrants exploration. Artificial intelligence enhancements and cost-effectiveness analyses would support scalability. Equity-focused research across diverse populations, particularly addressing recruitment barriers identified in this study, remains essential.

## 5. Conclusions

The P3-BC intervention represents a novel, theory-driven, web-based decision support system specifically designed to facilitate SDM about urinary diversion options for bladder cancer patients undergoing cystectomy. This feasibility study provides preliminary evidence that the P3-BC is feasible, usable by patients across a range of ages and computer experience, and acceptable to patients based on multiple dimensions of satisfaction and perceived value. Findings address a key gap in bladder cancer care, in which guidelines recommend SDM, but evidence-based tools are limited. Recruitment barriers (treatment burden, time constraints, and technology concerns) provide important guidance for future trials. Overall, these results support progression to adequately powered randomized controlled trials to evaluate clinical effectiveness and patient-centered outcomes.

## Figures and Tables

**Table 1:** Sociodemographic and clinical characteristics of patients with bladder cancer enrolled in the study who completed the baseline P3-BC survey (N = 15).

Characteristic	Total (N = 15)	Intervention (n = 10)	Control (n = 5)	*p*-Value
Age, mean (SD)	70.0 (7.1)	67.9 (3.1)	79.8 (4.1)	<0.001 †
Race, n (%)				0.43 ‡
White	13 (86.7)	9 (90.0)	4 (80.0)	
Black/African American	1 (6.7)	1 (10.0)	0	
Asian	1 (6.7)	0	1 (20.0)	
Ethnicity, n (%)				—
Hispanic	0	0	0	
Non-Hispanic	15 (100)	10 (100)	5 (100)	
Employment Status, n (%)				0.29 ‡
Work from home	1 (6.7)	1 (10.0)	0	
Part-time	2 (13.3)	2 (20.0)	0	
Full-time	1 (6.7)	1 (10.0)	0	
Retired	11 (73.3)	6 (60.0)	5 (100)	
Medical leave	1 (6.7)	1 (10.0)	0	
Unemployed	0	0	0	
Relationship Status, n (%)				0.62 ‡
Single	0	0	0	
Married	14 (93.3)	9 (90.0)	5 (100)	
Divorced	1 (6.7)	1 (10.0)	0	
Separated	0	0	0	
Widowed	0	0	0	
Gender, n (%)				0.10 ‡
Male	11 (73.3)	9 (90.0)	2 (40.0)	
Female	4 (26.7)	1 (10.0)	3 (60.0)	
Comorbidities, n (%)				
Chronic kidney disease	1 (6.7)	0	1 (20.0)	0.33 ‡
Heart disease	2 (13.3)	1 (10.0)	1 (20.0)	0.99 ‡
Diabetes	6 (40.0)	5 (50.0)	1 (20.0)	0.58 ‡
Peripheral neuropathy	1 (6.7)	1 (10.0)	0	0.99 ‡

Note. † Welch’s independent samples *t*-test; ‡ Fisher’s exact test (two-sided); — Not applicable (no variability).

**Table 2:** Program-Based acceptability and usability survey at 1-month follow-up (n = 10).

Scale Item Number	Program-Based Acceptability Measure (n = 10)	Low (%)	Neutral (%)	High (%)
1.	How understandable were the questions?	0	0	100
2.	How easy was the program to use?	0	14	86
3.	Overall satisfaction with the program	0	14	86
4.	Usefulness of information on statistics	14	0	86
5.	Was the amount of time to complete the program acceptable?	0	29	71
6.	Usefulness of information about top concerns	28	14	58
7.	How helpful was it to complete the program?	14	29	57
8.	How valuable was the information?	14	14	72
9.	Usefulness of videos	14	29	57
10.	Usefulness of information on the role in decision-making	14	29	57
11.	Usefulness of links to bladder cancer websites	0	17	83
12.	Usefulness of examples of what to say to your doctor	28	29	43

Note. Original responses (1–5 visual analog scale) were categorized as Low (scores = 1–2), Neutral (score = 3), or High (scores = 4–5). This approach preserves distinctions between endorsement and ambivalence and is consistent with best practices for feasibility and acceptability assessment in early-phase intervention studies.

**Table 3:** Survey-based acceptability and usability survey at 1-month follow-up (n = 8).

Scale ItemNumber	Survey-Based Acceptability Measure	Agree (%)	Disagree (%)
1.	Helped me make my treatment decisions	71.4	28.6
2.	Increased my knowledge about my treatment (diversion options)	62.5	37.5
3.	Made me feel less anxious or upset about my treatment	62.5	37.5
4.	Sort out what’s important to me for decision-making	62.5	37.5
5.	Taught me how to use stoma appliances or catheters	62.5	37.5
6.	Taught me how to care for myself after surgery	62.5	37.5
7.	Helped me reduce my anxiety about my treatment	62.5	37.5
8.	Helped me talk to doctors about my treatment options	50.0	50.0
9.	Helped me take care of myself	50.0	50.0
10.	Helped me manage my disease	50.0	50.0
11.	Prepared me for the surgery side effects	37.5	62.5

Note. Responses were categorized to agree (scores = 1–2 strongly agree/agree), or disagree (scores = 3–5; strongly disagree, disagree, I do not know).

**Table 4:** Differences between the intervention and control groups in study secondary outcomes at 1-month follow-up.

Outcomes at 1 Month	Group	N	Mean (SD)	Mean Difference (P3-BC–Control)	t(df)	*p*-Value	95% CI of Difference
Shared Decision-Making	P3-BC	10	5.43 (0.54)	−0.04	−0.14 (7.21)	0.889	−0.67, 0.60
	Control	4	5.47 (0.42)				
Brief Symptom Scale	P3-BC	8	0.51 (0.73)	0.36	1.27 (8.92)	0.238	−0.28, 1.00
	Control	3	0.15 (0.21)				

Note. Participants are included in the analyses if they completed relevant scales.

**Table 5:** Differences between the intervention and control groups in study outcomes at 3-month follow-up.

Outcomes at 3 Months	Group	N	Mean (SD)	Mean Difference (P3-BC–Control)	t(df)	*p*-Value	95% CI of Difference
Decisional Conflict	P3-BC	8	4.28 (0.39)	−0.28	−1.06 (3.61)	0.354	−1.05, 0.48
	Control	3	4.56 (0.39)				
Shared Decision-Making	P3-BC	8	4.28 (0.39)	−0.28	−1.06 (3.61)	0.354	−1.05, 0.48
	Control	3	4.56 (0.39)				
Brief Symptom Scale	P3-BC	8	0.26 (0.32)	0.19	1.54 (8.79)	0.160	−0.09, 0.47
	Control	3	0.07 (0.08)				
Decisional Regret	P3-BC	8	4.38 (0.59)	−0.29	−0.92 (5.29)	0.399	−1.10, 0.51
	Control	3	4.67 (0.42)				

## Data Availability

Data are available upon reasonable request. De-identified data used in this study will be made available upon reasonable request to the senior author (NM) following completion of an institutional data sharing agreement.

## Supplementary Materials

The following supporting information can be downloaded at: https://www.mdpi.com/article/10.3390/cancers18101501/s1, Figure S1: Study Conceptual Model; Figures S2 and S3: Overview of P3-BC System Interface; Table S1: Adaptation of the P3-P Intervention to Develop P3-BC Using the ADAPT-ITT Framework; Table S2: Application of ADAPT-ITT Framework to P3-BC Development.

## Abbreviations

The following abbreviations are used in this manuscript:

MIBC	Muscle-invasive bladder cancer
SDM	Shared decision making
ADAPT-ITT	Assessment, Decision, Adaptation, Production, Topical Experts, Integration, Training, Testing
AUA	American Urological Association
EAU	European Urology Association
ASCO	American Society of Clinical Oncology
IPDAS	International Patient Decision Aid Standards
P3-P	Personal Patient Profile–Prostate Cancer
P3-BC	Personal Patient Profile–Bladder Cancer
BCAN	Bladder Cancer Advocacy Network
UOAA	United Ostomy Association of America
