# Supplementary material for: Development and Evaluation of the Personal Patient Profile–Bladder Cancer (P3-BC): A Web-Based Decision Support System for Patients Considering Cystectomy and Urinary Diversion
Source: Cancers (Basel). 2026 May 7;18(10):1501. doi: 10.3390/cancers18101501 (PMC13204023; doi:10.3390/cancers18101501)
Supplement: Supplementary file 1 [file cancers-18-01501-s001.zip › cancers-4236974-supplementary.pdf]

Supplementary Table S1. *Adaptation of the P3-P Intervention to Develop P3-BC Using the ADAPT-ITT Framework*

**Intervention Domain**	**P3-P (Original Intervention)**	**Adaptation Decision**	**P3-BC (Bladder Cancer–Specific Adaptation)**
Tailoring Algorithm	Assessment-driven tailoring algorithm guiding content delivery based on patient preferences and values	Retained	Core algorithm structure preserved to maintain fidelity; logic updated to incorporate bladder cancer–specific decisional needs
Values Clarification	Exercises focused on tradeoffs among treatment efficacy, side effects, and lifestyle impact	Modified	Reframed to address body image, urinary control methods, self-care burden, physical activity, and adaptation to altered bodily function
Treatment Options	Binary or limited multiple treatment choices	Modified	Expanded to include three urinary diversion options: ileal conduit, continent cutaneous diversion, and orthotopic neobladder
Outcome Presentation	Emphasis on cancer control and treatment-related side effects	Modified	Shifted focus to functional outcomes, self-care requirements, lifestyle adaptation, long-term complications, and quality of life
Multimedia Content	Text, graphics, and videos illustrating treatment concepts	Modified	New videos demonstrating stoma care, catheterization techniques, appliance management, and daily self-care routines
Communication Coaching	Coaching prompts to support patient–clinician discussion	Retained	Communication coaching approach preserved; prompts tailored to diversion-specific questions and concerns
Probabilistic Information	Graphical display of risks and benefits	Retained	Graphical formats preserved; probabilities updated to reflect diversion-specific outcomes and complications
Assessment Queries	General cancer treatment concerns and preferences	Modified	Expanded to capture bladder cancer–specific concerns (e.g., stoma management, catheterization, leakage, body image)
User Interface Design	User-centered, touchscreen-based interface	Retained	Interface principles maintained to ensure accessibility, usability, and engagement

Self-Care Education	Limited post-treatment self-management content	New Content	Detailed stoma care instructions, catheterization schedules, appliance management guidance
Physical Activity Guidance	Not included	New Content	Guidance on resuming physical activity and exercise tailored to each diversion type
Sexual Function	Minimal coverage	New Content	Information on sexual function impacts and adaptation strategies by diversion type
Long-Term Complications	General treatment risks	New Content	Diversion-specific long-term complication education and monitoring guidance

Supplementary Table S2. *Application of ADAPT-ITT Framework to P3-BC Development*

**ADAPT-ITT Phase**	**Study Activities**	**Key Outcomes**
**Phase 1: Assessment**	Focus groups and interviews with MIBC patients (n=30); literature review; analysis of prior cystectomy patient data	Identified key concerns: catheterization, stoma, body image, functional impacts; documented decisional priorities and information needs
**Phase 2: Decision**	Review of existing P3-P intervention; assessment of fit for bladder cancer context; multidisciplinary team review	Decision to adapt P3-P for bladder cancer; identified core components to retain (tailoring, values clarification, multimedia) and areas requiring modification (content, options, outcomes)
**Phase 3: Adaptation**	Systematic comparison of prostate vs. bladder cancer decision contexts; identification of necessary modifications; development of adaptation matrix	Documented differences: multiple reconstruction options vs. treatment choice; adaptation to living with new bodily function vs. treatment of existing organ; emphasis on self-care and lifestyle adaptation

**Phase 4: Production**	Content development; creation of new text, graphics, videos; programming of tailoring algorithm; interface design	Complete P3-BC application with bladder cancer-specific content, updated tailoring queries, and technical platform
**Phase 5: Topical experts**	Consultation with urologic oncologists, ostomy nurses, health informaticists, patient advisors; patient advocates from BCAN and UOAA, content validation; clinical guideline review	Evidence-based, clinically accurate content aligned with current guidelines; patient-centered language and framing
**Phase 6: Integration**	Synthesis of all stakeholder input; refinement of content and interface; documentation of core vs. adapted components	Integrated P3-BC intervention maintaining fidelity to P3 approach while addressing bladder cancer-specific needs
**Phase 7: Training**	Prepilot usability testing with "think-aloud" protocol (n=10 stakeholders); identification of navigation and content issues; staff training on technical support	Refined navigation, clarified medical terminology, corrected technical issues; staff prepared for pilot testing
**Phase 8: Testing**	Clinical pilot with MIBC patients (n=15); assessment of feasibility, acceptability; systematic data collection	Demonstrated feasibility and high acceptability (86% satisfaction)

Figure S1: Study Conceptual Model

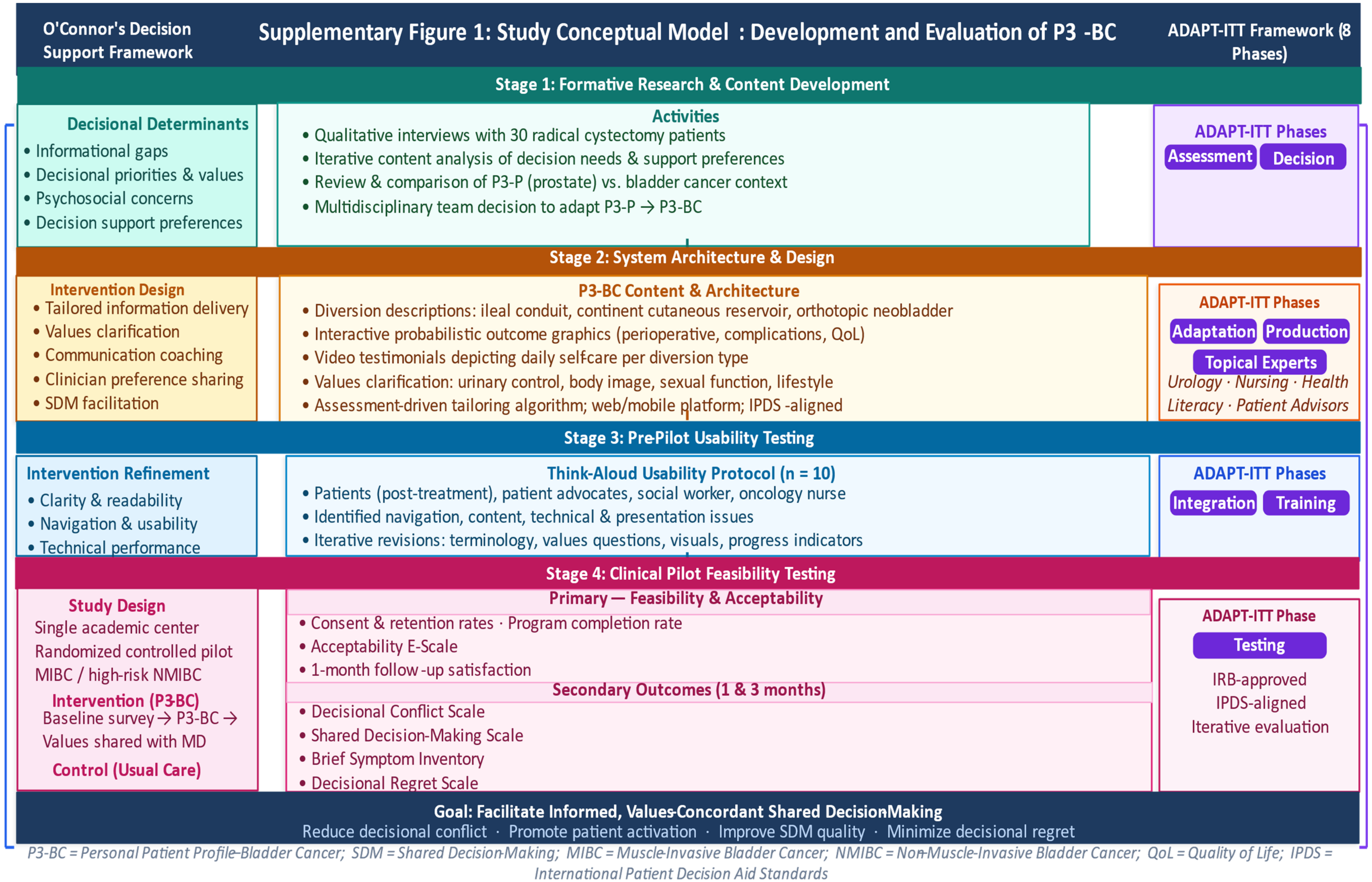

P3-BC = Personal Patient Profile-Bladder Cancer; SDM = Shared Decision-Making; MIBC = Muscle-Invasive Bladder Cancer; NMIBC = Non-Muscle-Invasive Bladder Cancer; QoL = Quality of Life; IPDS = International Patient Decision Aid Standards

Figures S2–S3: Overview of P3-BC System Interface

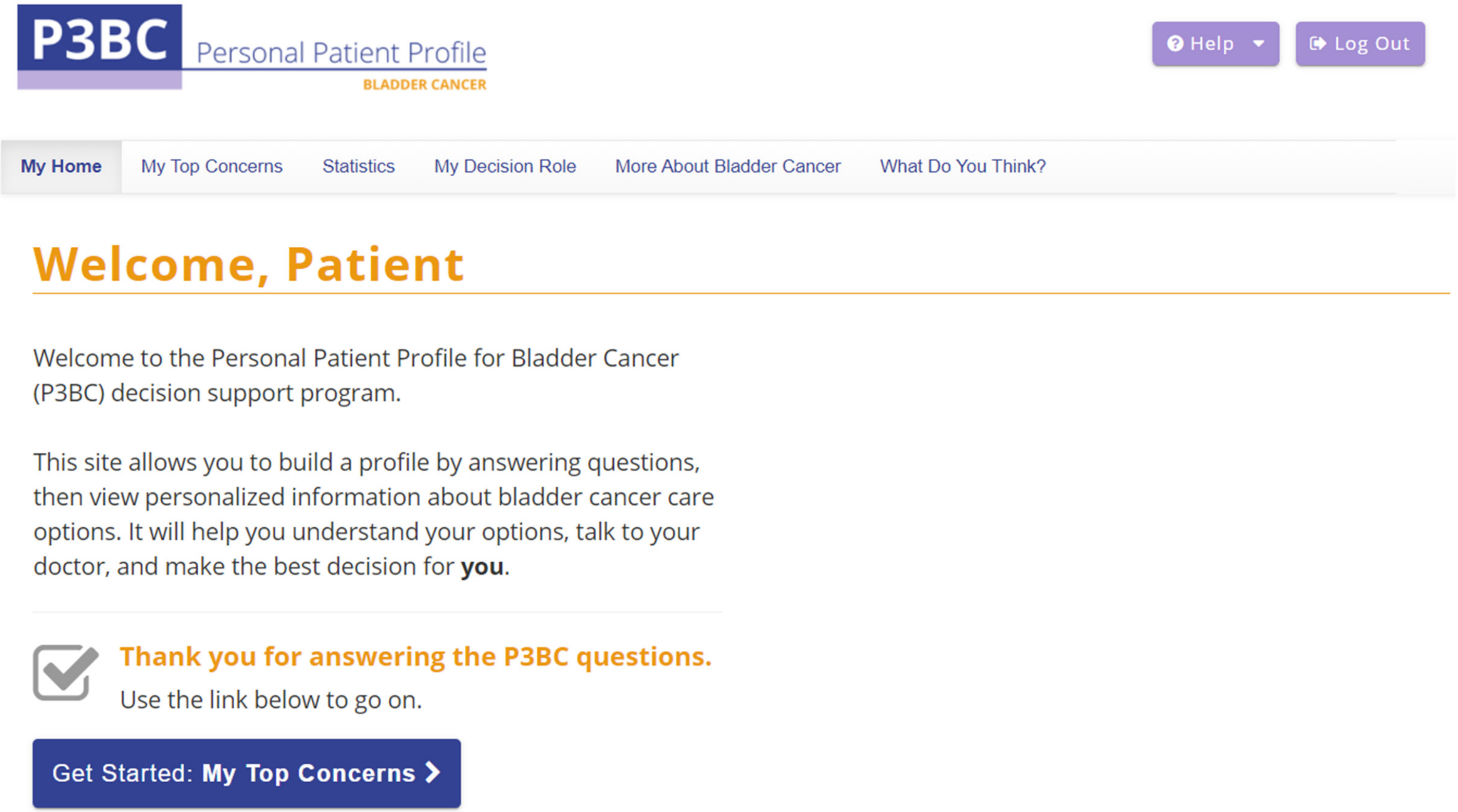

P3BC Personal Patient Profile
BLADDER CANCER

Help | Log Out

My Home | My Top Concerns | Statistics | My Decision Role | More About Bladder Cancer | What Do You Think?

Click to see other approaches:
- Decision Is Yours
- Decision Is Shared
- Decision Is Doctor's

My Role in Making a Decision

Talk to your doctor about the role you feel comfortable taking in making a decision about your care. This will help your doctor understand what you want.

Based on your answers, your preferred way to make a decision about your reconstruction decision is shown below. You can also learn about other ways of making a decision by using links on the left to watch other videos.

DECISION IS SHARED

Your preferred way to make a decision about bladder reconstruction surgery:

> **I prefer that my doctor(s) and I share the decision about which option is best.**

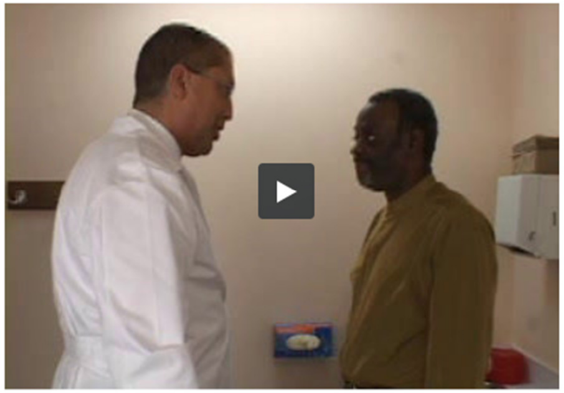

Next Section: **More About Bladder Cancer**

Figures S2–S3. *P3-BC home screen ('My Home').*

The P3-BC welcome screen provides patients with an orientation to the program. The header displays the P3BC Personal Patient Profile – Bladder Cancer branding with navigation options for Help and Log Out. The main navigation bar provides access to all program modules: My Home, My Top Concerns, Statistics, My Decision Role, More About Bladder Cancer, and What Do You Think? The welcome message explains the program's purpose — to help patients build a personal profile, view personalized information about bladder cancer care options, understand their options, talk to their doctor, and make an informed decision. A prominent call-to-action button ('Get Started: My Top Concerns') guides patients into the first content module. The program is accessible via desktop computer and smartphone.
